# Early Diagnosis of Oral Cancer and Lesions in Fanconi Anemia Patients: A Prospective and Longitudinal Study Using Saliva and Plasma

**DOI:** 10.3390/cancers15061871

**Published:** 2023-03-21

**Authors:** Ricardo Errazquin, Estela Carrasco, Sonia Del Marro, Anna Suñol, Jorge Peral, Jessica Ortiz, Juan Carlos Rubio, Carmen Segrelles, Marta Dueñas, Alicia Garrido-Aranda, Martina Alvarez, Cristina Belendez, Judith Balmaña, Ramon Garcia-Escudero

**Affiliations:** 1 Research Institute Hospital 12 de Octubre (Imas12), University Hospital 12 de Octubre, Av Cordoba s/n, 28041 Madrid, Spain; 2 Molecular Oncology Unit, CIEMAT (Centro de Investigaciones Energeticas, Medioambientales y Tecnologicas), Avenida Complutense 40, 28040 Madrid, Spain; 3 Hereditary Cancer Genetics Group, Medical Oncology Department, Vall d’Hebron Institute of Oncology (VHIO), 08035 Barcelona, Spain; 4 Centro de Investigación Biomédica en Enfermedades Raras (CIBERER), Instituto de Salud Carlos III, 28029 Madrid, Spain; 5 Centro de Investigación Biomédica en Red de Cáncer (CIBERONC), Instituto de Salud Carlos III, 28029 Madrid, Spain; 6 Centro de Investigaciones Médico-Sanitarias (CIMES), 29071 Malaga, Spain; 7 Sección de Hematología y Oncología Pediátricas, Hospital General Universitario Gregorio Marañón, 28007 Madrid, Spain; 8 Facultad de Medicina, Universidad Complutense de Madrid, 28040 Madrid, Spain; 9 Instituto Investigación Sanitaria Gregorio Marañón, 28007 Madrid, Spain

**Keywords:** Fanconi anemia, head and neck squamous cell carcinoma, oral squamous cell carcinoma, OSCC, SCC, oral potentially malignant lesion, OPML, leukoplakia, precursor lesion, saliva, plasma, *TP53*, mutation, next generation sequencing, cancer gene

## Abstract

**Simple Summary:**

Patients with Fanconi anemia (FA) have a very high risk of developing oral lesions and squamous carcinomas at early ages. As treatment strategies in this setting are very limited, new early diagnosis methods are urgently needed. We performed a pilot, prospective clinical study in which saliva and plasma samples were analyzed with the deep sequencing of cancer genes. The patients included had apparently normal oral mucosa when recruited. Mutations were detected in the liquid biopsies with allele frequencies of down to 0.07%. We found that patients with mutations displayed a higher risk of developing lesions/carcinomas after mutation detection. We propose that this non-invasive, highly sensitive technology could allow for the better management of these pathologies in FA individuals.

**Abstract:**

Fanconi anemia (FA) patients display an exacerbated risk of oral squamous cell carcinoma (OSCC) and oral potentially malignant lesions (OPMLs) at early ages. As patients have defects in their DNA repair mechanisms, standard-of-care treatments for OSCC such as radiotherapy and chemotherapy, give rise to severe toxicities. New methods for early diagnosis are urgently needed to allow for treatment in early disease stages and achieve better clinical outcomes. We conducted a prospective, longitudinal study wherein liquid biopsies from sixteen patients with no clinical diagnoses of OPML and/or OSCC were analyzed for the presence of mutations in cancer genes. The DNA from saliva and plasma were sequentially collected and deep-sequenced, and the clinical evaluation followed over a median time of approximately 2 years. In 9/16 FA patients, we detected mutations in cancer genes (mainly *TP53*) with minor allele frequencies (MAF) of down to 0.07%. Importantly, all patients that had mutations and clinical follow-up data after mutation detection (*n* = 6) developed oral precursor lesions or OSCC. The lead-time between mutation detection and tumor diagnosis ranged from 23 to 630 days. Strikingly, FA patients without mutations displayed a significantly lower risk of developing precursor lesions or OSCCs. Therefore, our diagnostic approach could help to stratify FA patients into risk groups, which would allow for closer surveillance for OSCCs or precursor lesions.

## 1. Introduction

Head and neck squamous cell carcinoma (HNSCC) arises in the mucosal lining of the upper aerodigestive tract, which includes the oral cavity, larynx and pharynx [1]. Carcinomas in the oral cavity can evolve from visible oral potentially malignant lesions (OPMLs) such as leukoplakias and erytroplakias (white or red patches, respectively) [2]. The clinical outcomes of OPMLs are variable, with many lesions that regress. Risk assessment methods are based on histopathology analyses from tissue biopsies, but these can fail as some low-risk lesions could become malignant [3]. The development of more accurate prediction methods, possibly including genetic analyses such as the loss of heterozygosity (LOH), is needed [4]. Furthermore, lesions can be treated with surgery [5], but even when margins are negative, recurrence rate is high, demonstrating that active treatment does not always prevent OSCC [6]. These recurrent tumors might arise from mutant precancerous fields that display normal histology [7]. Occasionally, patients have large or multifocal lesions that impede treatment by resection. Therefore, the frequent surveillance of OPMLs might be an alternative until new curative therapies are developed [8]. On the other hand, many patients display tumors *de novo* with no previous clinical symptoms. These tumors originate from invisible premalignant cells, possibly within precancerous fields [7]. Currently, there are no tools to detect such cells before carcinoma development, thus hampering the appropriate clinical management of OSCC patients.

Risk factors for OSCC and OPMLs include alcohol and tobacco consumption, as well as genetic predisposition. Fanconi anemia (FA) is a genetic syndrome with a propensity to congenital malformations, bone marrow failure (BMF) and cancer [9,10]. Most FA children develop BMF which is treated with a hematopoietic stem cell transplantation (HSCT), significantly improving life expectancy. Current advances in a gene therapy clinical trial might provide an alternative to HSCT in the near future for children with FA who may potentially develop BMF [11]. Sadly, HNSCC propensity is currently the most important health challenge in FA patients, with incidences 500 times higher than the general population [12]. OSCC in FA is associated with a high mortality rate. The median age at diagnosis is around 25–31 years old, half of the age of standard patients. Pathogenic mutations have been reported in 23 different genes that give rise to Fanconi or a Fanconi-like clinical phenotype [9,10]. A hallmark feature of FA is the inability to repair DNA interstrand cross-links (ICLs). FA proteins repair ICLs in a common cellular pathway known as the FA pathway or FA/BRCA pathway. HNSCC in FA is difficult to treat, as patients cannot tolerate current therapies, which include ionizing radiation and platinum-based chemotherapies [13]. In addition, most tumors are detected at advanced stages. Altogether, these events have made cancer the principal cause of early mortality in adults with FA. Therefore, the discovery of non-genotoxic and efficient treatment opportunities for FA patients is of the utmost importance.

FA individuals develop OPMLs, mainly leukoplakias, at much higher rates than the general population: 9–12% versus 1–2%, respectively [14,15,16]. Furthermore, the average age at presentation is much younger: 15–16.5 years old in FA patients versus 45–54 years old in non-FA patients. Oral lesions in FA are clinically challenging, as they frequently involve large areas and appear multifocally. Patients display consecutive lesions which recur frequently and can progress to malignant carcinomas. Importantly, FA individuals with leukoplakias are at higher risk of OSCC. Close surveillance is important and should begin at pediatric ages, especially after HSCT. Therefore, there is an urgent clinical need for new technologies that enable the accurate detection of premalignant cells. Due to close and repeated examination, a noninvasive technique would be desirable as tissue biopsies are a burden for the patient.

Liquid biopsies (LBs) constitute a non-invasive source of patient biological material that can help to diagnose pathologies such as cancer. LBs can be collected easily and repeatedly during pathologies that require close surveillance. Methods to detect cancer biomarkers in LBs, such as tumor DNA, have substantially improved over the last decade. Thus, some technologies based on next-generation sequencing (NGS) can detect tumor DNA molecules with limit of detection (LOD) of <0.1%. This high sensitivity has been particularly useful when analyzing circulating tumor DNA (ctDNA) in plasma. The detection of ctDNA provides real-time knowledge of cancer, beginning with screening before it is clinically apparent [17] and during all stages of cancer treatment [18]. Importantly, LBs have already entered clinical practice, guiding treatment in a subset of lung tumors. In addition to plasma, another LB with a potential utility for cancer research is saliva. In HNSCC patients, the existence of tumor cells and tumor-derived DNA (tDNA) in saliva has been reported [19]. As a tumor grows, it releases tDNA into saliva so that the mutations found in tDNA are equal to those of the primary tumor [20,21]. Therefore, tDNA could be used as a surrogate for a tissue biopsy. Indeed, Wang et al. reported that saliva and plasma analysis allowed for the detection of somatic mutations and human papillomavirus (HPV) in 96% of HNSCC patients (100% in oral cavity) [21], showing that the evaluation of plasma can complement that of saliva. To our knowledge, the utility of tumor DNA detection in saliva and plasma for the early diagnosis of OSCC in high-risk patients has not been tested.

Here, we present a proof-of-principle study wherein the NGS-based, sensitive detection of cancer DNA mutations in saliva and plasma from FA patients could help to detect OPMLs and/or OSCC before clinical diagnosis. LBs from patients were repeatedly collected in a prospective and longitudinal clinical study of approximately 2 years. The results demonstrated that our biomarker approach could help to identify FA patients at a very high risk of OPMLs and OSCC.

## 2. Materials and Methods

### 2.1. Study Design and Patient Cohort

This was a multi-center, non-interventional, prospective longitudinal cohort clinical study. Fanconi anemia patients with no previous confirmed evidence of the development of solid or blood malignancies were considered eligible. Patients with myelodysplastic syndrome were excluded, as well as those with major health issues. Sixteen Spanish patients were recruited at the Hospital Universitario Gregorio Marañon (Madrid, Spain) and the Hospital Universitario Vall d’Hebron (Barcelona, Spain) between November 2018 and December 2021. The primary objective was the detection of mutations in cancer genes, using the DNA from liquid biopsy samples prior to the clinical diagnosis of lesions or carcinomas in the head and neck. We included HSCT patients older than 14 years old (*n* = 11) and non-HSCT patients older than 25 years old (*n* = 5). Clinical follow-up at the Oral Dentistry Department was conducted as part of the clinical surveillance. All patients provided their consent by signing a form appropriate to the study, which was approved by the Ethics Committees for Clinical Research of the hospitals involved. Saliva and whole blood were collected from each patient at least once during the duration of the project, and yearly whenever possible. Sample/patient IDs are not known to anyone outside the research group.

### 2.2. Saliva and Plasma Collection and DNA Purification

Saliva samples were collected as oral rinses. Patients were asked to swish 10 mL of 0.9% sodium chloride in their mouths for 10 to 15 s before spitting into the collection tube. Venous blood (4–10 mL) was collected using standard phlebotomy techniques in EDTA tubes and maintained at 4 °C until further processing. Plasma was obtained from blood within 4 h of phlebotomy upon two sequential centrifugations: the first at 1200× *g* for 7 min at 4 °C and the second at 14,000× *g* for 10 min at 4 °C. In some instances, the buffy coat layer was recovered during blood processing, lysed using a BD FACS Lysing Solution (Thermo Fisher Scientific, Waltham, MA, USA), centrifuged at 3000 rpm for 10 min, and stored at −80 °C. The saliva genomic DNA (gDNA) was extracted from 400 μL of oral rinses using a MagMAX Saliva gDNA Isolation Kit (Thermo Fisher Scientific, Waltham, MA, USA). Plasma-circulating cell-free DNA (cfDNA) was extracted from 4 mL of plasma using a MagMAX Cell-Free Total Nucleic Acid Isolation Kit (Thermo Fisher Scientific, Waltham, MA, USA). Leucocyte DNA was extracted using a DNeasy Blood & Tissue Kit (Qiagen, Hilden, Germany). DNA was quantified using Qubit Fluorometric Quantification (Thermo Fisher Scientific, Waltham, MA, USA).

### 2.3. Library Preparation and Deep-Sequencing

Libraries for next-generation sequencing (NGS) were constructed from 10–50 ng of DNA, using the Oncomine^TM^ Pan-Cancer Cell-Free Assay (OPA) (Thermo Fisher Scientific, Waltham, MA, USA). They were quantified using the Ion Library TaqMan^TM^ Quantification Kit (Thermo Fisher Scientific, Waltham, MA, USA). Templating and sequencing were performed using the Ion 540™ sequencing chip on the Ion Chef and Ion S5 XL systems (Thermo Fisher Scientific, Waltham, MA, USA), according to the manufacturer’s instructions. Although the OPA is designed to detect some gene fusions from RNA, we did not analyze them.

### 2.4. Sequencing Analysis

Sequencing raw data were analyzed on the Torrent Server^TM^, reads were aligned to the reference human genome (hg19), and a variant analysis was performed using the Ion Reporter^TM^ Analysis Server. Quality control was performed manually for every sample based on the following filters: number of mapped reads per sample >10,000,000; and on-target reads >90%. For samples with missing data on these filters, we filtered out those with a median molecular coverage <500. Median molecular coverage is the median number of individual, interrogated DNA molecules across targets. We always require two independent molecular families to identify a variant. Variant calling was performed with the Ion Reporter Analysis Software v.5.18, using the Oncomine TagSeq Pan-Cancer Liquid Biopsy w.2.5-Single Sample workflow and the predefined filter chains Variant Matrix Summary (5.18) and Oncomine Extended (5.18). Only variants with at least two molecular counts and with a minor allele frequency (MAF) of >0.065% were included in the analysis. MAF is the mutant allele proportion among the total molecular number (consisting of both wild-type and mutated sequences), expressed as a percentage. We included copy number variants (CNVs) with a ratio above 1.15 and with a median absolute pairwise difference (MAPD) below 0.4. Using these thresholds, we did not detect CNVs in our LB samples. Known germline variants with an allelic frequency above 35% or single-nucleotide polymorphisms were not included in the analysis. Leucocyte DNA from some FA patients was similarly sequenced and analyzed.

### 2.5. Survival Curves

Kaplan–Meier survival curves were obtained with Prism software, v.9.0 (Graphpad Software, Inc., San Diego, CA, USA). The statistical significance of survival between genotypes was calculated with the log-rank test, yielding a *p*-value.

## 3. Results

Patients’ characteristics. A total of 16 Fanconi anemia patients from two clinical centers in Spain (Hospital Vall d’Hebron in Barcelona and Hospital Gregorio Marañon in Madrid) were included in the study. These patients were checked each year at the Departments of Hematology, Maxillofacial and Hereditary Cancer. At the time of enrollment, patients had not had previous clinical diagnoses of OPML and/or OSCC. However, we cannot discard the existence of microscopic lesions or carcinomas. We included HSCT patients older than 14 years old (*n* = 11) and non-HSCT patients older than 25 years old (*n* = 5). Patient characteristics, clinical follow-up times, lesions and cancer outcomes are detailed in Table 1 and Supplementary Table S1.

**Figure 1:**
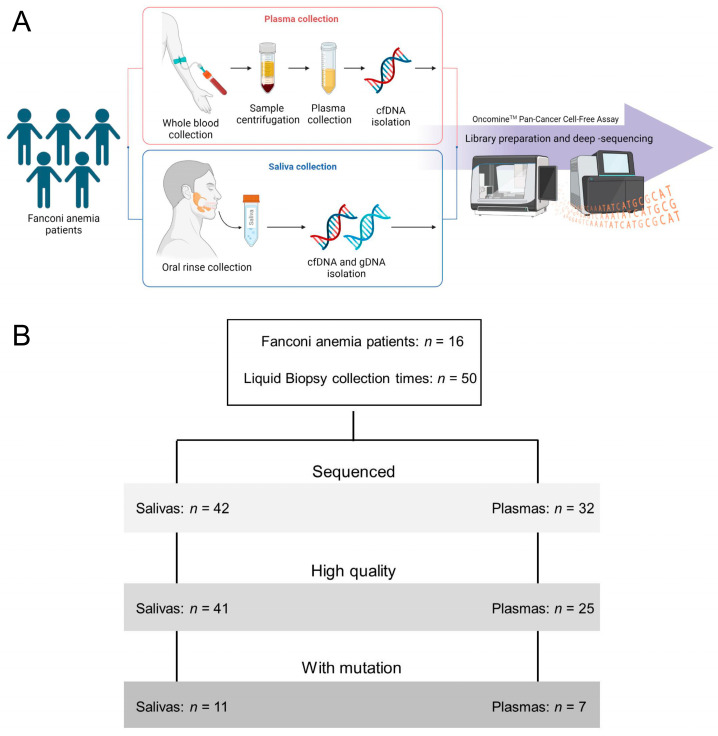
(**A**) Blood and oral rinses were longitudinally collected from FA patients with no clinical diagnoses of OPML and/or OSCC. DNA was purified and deep-sequenced using the Oncomine^TM^ Pan-Cancer Cell-Free Assay (OPA) from Thermofisher. Bioinformatics analysis was performed to detect the presence of mutations in cancer genes in the liquid biopsies. (**B**) There was a total of 50 collection times of liquid biopsies from 16 FA patients during the clinical study. A subset of samples was deep-sequenced with the OPA assay. Only high-quality sequencing runs were analyzed for the presence of cancer mutations.

**Figure 2:**
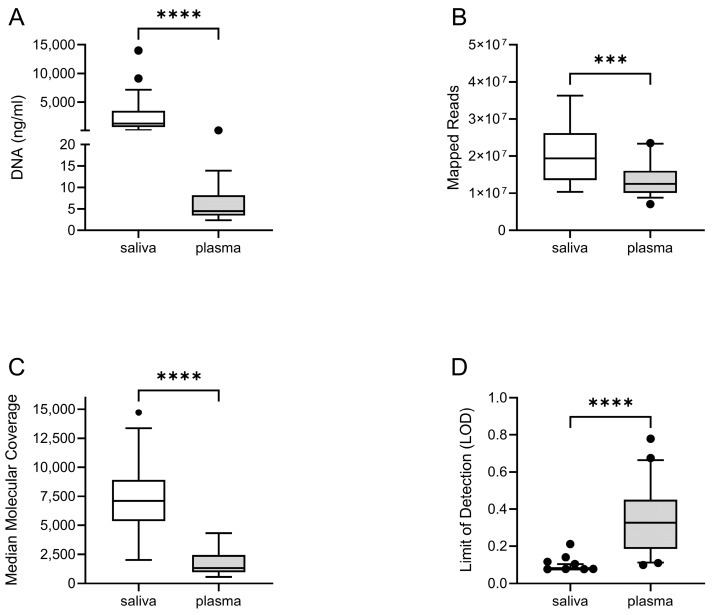
Sequencing performance in saliva and plasma. (**A**) The concentration of DNA purified from saliva was significantly higher than that obtained in plasma, as expected. The total number of mapped reads (**B**) and the median molecular coverage (**C**) showed a better sequencing performance of saliva versus plasma. Consequently, the median limit of detection (LOD %) across targets was lower in saliva (**D**), thus allowing detection of mutations at reduced frequencies in saliva to be more likely. ***: *p*-value < 0.001; ****: *p*-value < 0.0001.

Liquid biopsies collected. Saliva and/or plasma were collected at different time points during the longitudinal study (*n* = 50) (Figure 1). The DNA was purified and quantified from 42 saliva and 32 plasma samples, using dedicated methods (see M&M). As expected, the amount of DNA was much higher in saliva than plasma (Figure 2A). Patient genetic material in saliva arose from desquamated keratinocytes during oral rinses, and could potentially contain precancerous or tumor DNA (tDNA). In plasma, purified DNA is cell-free DNA (cfDNA), which normally exists in low quantities in tumor-free patients.

Sequencing performance in saliva and plasma. DNA from a LB was utilized for library preparation and quantification using the Oncomine^TM^ Pan-Cancer Cell-Free Assay (OPA). OPA analysis allows for the detection of molecular aberrations in 52 genes that are frequently mutated in solid tumors (Supplementary Table S2). Deep-sequencing was performed using the Ion Chef™ and Ion S5 XL systems (Thermo Fisher Scientific, Waltham, MA, USA). An initial analysis of performance demonstrated that the sequencing was optimal for most of the LB samples (Figure 1), mainly for saliva samples. The number of mapped reads and the median molecular coverage (median number of individual interrogated DNA molecules across targets) allowed for limit of detection (LOD) values as low as 0.07% in saliva and 0.09% in plasma (Figure 2B–D). The LOD values were more variable in plasma samples as the DNA yield obtained was below 20 ng in most cases (Supplementary Table S3).

Mutations found in liquid biopsies. Although the OPA assay is designed to detect copy number variants (CNVs) in 12/52 genes (Supplementary Table S2), we did not find CNVs in any LB sample. Importantly, we found non-synonymous small variants or mutations (SNVs, MNVs and INDELs) in 15 samples from 9 patients (Supplementary Table S4). As mutations are found at a low MAF, we could not discard the possibility that they arose from small populations of leucocytes via a selection process such as clonal hematopoiesis (CH). Therefore, we performed the OPA assay using leucocyte DNA obtained from the blood of some of these patients (Supplementary Tables S3 and S4). We discarded one sample because of poor sequence quality (FA452) (see M&M). We found a mutation in *IDH2* that was also detected in the saliva of patient FA531. Considering that the MAF observed was below 0.1% in both cases, we suggest that this a mutation associated to CH and not to a lesion/SCC in the oral cavity. We also detected a mutation in *PDGFRA* that was not found in the LBs from FA145, so we did not discard any mutation in this patient.

**Figure 3:**
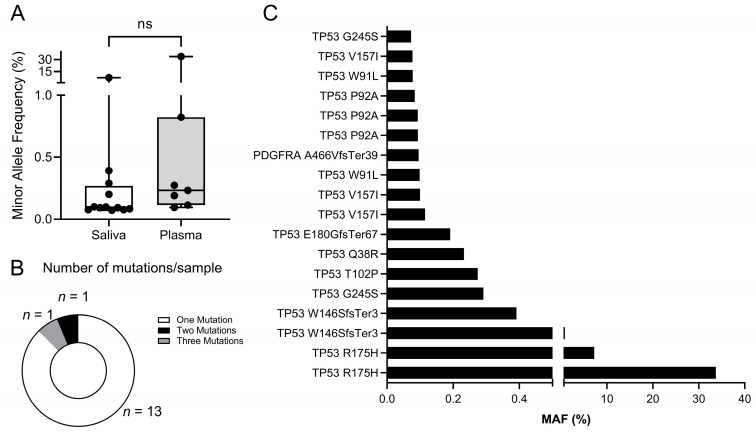
Mutations found in saliva and plasma. (**A**) A total number of 18 mutations (SNVs, MNVs and INDELs) were found in 15 samples, with similar minor allele frequencies (MAF %) between saliva and plasma. (**B**) Number of mutations per sample. Samples normally had single mutations (*n* = 13), although multiple concurrent mutations were also found. (**C**) Minor allele frequencies (MAF %) of mutations found. *TP53* was the most frequently mutated gene (95% of samples).

The minor allele frequencies (MAF %) of the mutations observed were similar between the saliva and plasma, with minimum values of around 0.07% (Figure 3A). A majority of mutant samples displayed only one mutation, but two samples had more than one mutation (Figure 3B). Interestingly, most mutations were mapped in the *TP53* gene (Figure 3C), which is in line with previous reports that demonstrated the high frequency of *TP53* mutations in HNSCC from FA patients [22,23].

**Figure 4:**
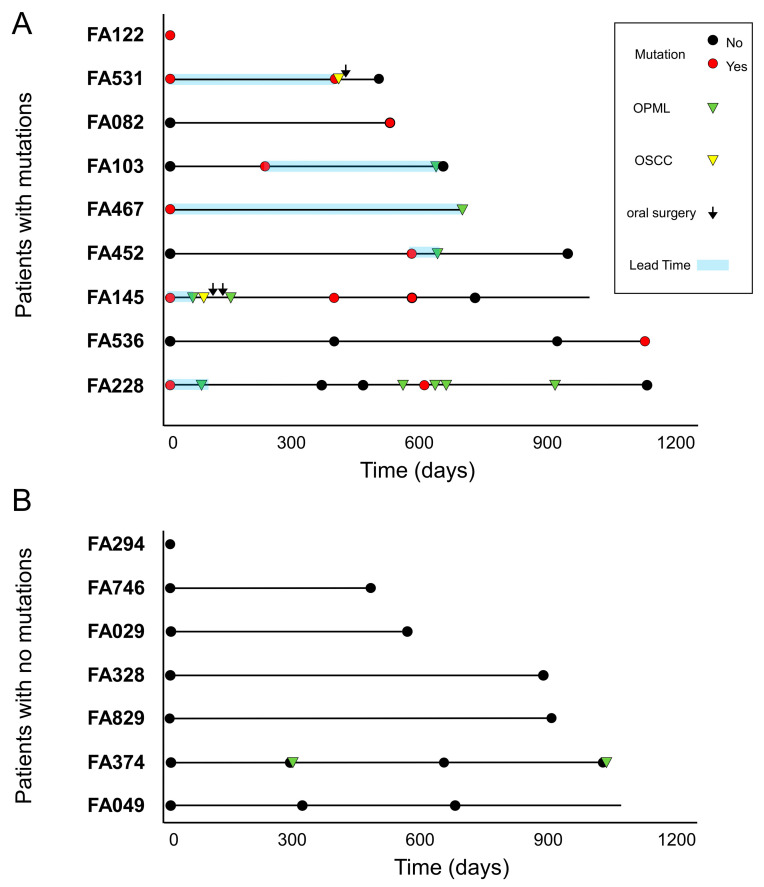
Fanconi anemia patients with mutations develop OPML and/or OSCC. Schematic representation of the molecular and clinical events during patient’s follow-up. (**A**) A total of 9/16 patients had mutations in at least one liquid biopsy sample, and 6/9 patients had clinical follow-up data after mutations were found; all of them displayed OPML or OSCC afterwards. Time from mutation detection to tumor detection (lead time) is shown (blue boxes). Solid horizontal lines represent clinically informed follow-up times from the first LB analyzed in each patient. (**B**) A total of 7/16 patients displayed no mutations, 6 of whom were free of OPML or OSCC.

Association between cancer mutations and clinical outcome. Fourteen FA patients from our cohort had a clinical follow-up after the collection of at least one LB. The median follow-up time from the first LB collection was 719 days. There were 7/14 patients who developed lesions or carcinomas in the oral region, and 7/14 patients who did not (Figure 4). Most lesions were leukoplakia in the oral cavity (Supplementary Table S5). Two patients had oral squamous cell carcinomas that were subsequently treated with surgery: FA531 and FA145.

**Figure 5:**
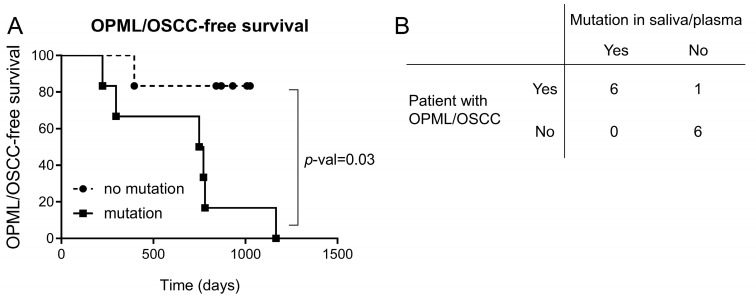
(**A**) Kaplan–Meier curves of FA patients with or without mutations. FA patients with mutations display significantly higher risk of developing OPML or OSCC than patients without mutations. Statistically significance differences between curves were determined using log rank test. (**B**) Accuracy of the detection of mutations in saliva/plasma as diagnostic for FA patients that develop OPML or OSCC. Global accuracy: 92%; sensitivity: 86%; specificity: 100%.

We found that 6/7 patients with lesions or tumors had at least one LB sample with a mutation in a cancer gene (Figure 4A). Importantly, we detected such mutations before clinical diagnosis, with lead times ranging between 23 and 630 days (median: 205 days). On the other hand, we could not find mutations in any of the LB samples from five patients with no previous diagnosis of OPML and/or OSCC (Figure 4B). Patient FA374 had two leukoplakias during the study, but we could not detect any mutation. No biopsies were taken from these lesions, and therefore no histopathology assessment was conducted (Supplementary Table S5). A Kaplan–Meier curve showed a significant difference in oral lesion/tumor-free survival between FA patients with a mutation versus patients with no mutations (Figure 5A). The accuracy of the diagnostic test to distinguish between patients that will develop oral lesions or carcinomas from those that will not is 92%, with a sensitivity of 86% and 100% specificity (Figure 5B). In conclusion, these results showed that the presence of a mutation in saliva or plasma in FA patients is predictive of the appearance of lesions/tumors in the oral cavity region and can distinguish between high- and low-risk groups.

## 4. Discussion

The mutation analysis of saliva and plasma enabled the stratification of Fanconi anemia patients into groups with significantly different risks of developing lesions or squamous carcinomas in the oral cavity. Our proof-of-principle approach involved noninvasive technologies that can be used repeatedly during the surveillance of patients to help detect premalignant or malignant cells before clinical symptoms appear. In addition, other cohorts with a high risk of OPMLs and OSCC, such as smokeless tobacco users, long-time smokers and heavy alcohol drinkers, might benefit from this technology.

More than 60 years ago, Szilard proposed that accumulation of mutations in somatic cells could accelerate aging in living organisms [24]. Indeed, a number of important reports using sensitive NGS technologies have confirmed the correlation between the somatic mutation index and aging in different animal species and organs, such as skin [25] or the esophagus [26]. Thus, mutagens such as UV light or tobacco increase mutation rates in cells (in vivo and in vitro) and accelerate senescence, apoptosis and the emergence of cancerous cells. Importantly, most genetic syndromes that impair normal DNA repair mechanisms (such as Fanconi anemia) are associated with premature aging and a predisposition to cancer [27]. Some of these syndromes display a high mutational burden in tissues in which the cancer risk is also higher [28]. Therefore, it is tempting to speculate that tissues from FA patients display an exacerbated accumulation of genomic aberrations which eventually might speed the generation of premalignant and malignant cells. Our results showing that patients with mutations in liquid biopsies are at higher risk of developing oral lesions or squamous carcinomas are compatible with the reported link between increased mutational burden and cancer risk. We hypothesize that some FA patients might have a somatic mutation index higher than other FA patients, and that index could be related to precancer or cancer risk. Future experiments with more sensitive methods and more FA patients in the context of a clinical study should help to validate our hypothesis.

*TP53* is the gene most frequently mutated in OSCC, both in FA patients [23,29] and in the general population [30]. In addition, *TP53* mutations have been detected in the early stages of OSCC, as shown in cellular model of precancer occur early derived from macroscopically normal mucosa of the surgical margins of patients with primary OSCCs [31]. Importantly, the *TP53* gene is the most frequently mutated gene in leukoplakias, mainly in lesions with dysplasia versus lesions with no dysplasia [32]. Therefore, *TP53* mutations could be detected in apparently normal oral mucosa from patients using highly sensitive methods if precancerous or cancerous cells already exist in the mucosa. Our results indicating the presence of *TP53* mutations in saliva and plasma in patients that developed lesions or carcinomas afterwards suggest that our method can detect such cells before they are clinically visible. Similar results have been reported using NGS in brush biopsies from locations of the oral cavity without visible abnormalities in FA patients. In line with our survival results (Figure 5A), patients with mutations (frequently in the *TP53* gene) had a higher risk of OSCC than patients with no mutations [32]. Unfortunately, we could not study whether the mutations found in LBs were also present in the consecutive lesion/tumor.

Recent next-generation sequencing analyses have demonstrated that, in addition to *TP53*, other genes such as CDKN2A and NOTCH1 display small variants both in OSCC tumors and in oral brush biopsies from Fanconi anemia patients [23,32]. Unfortunately, none of these genes are included in the OCA panel that we used, and we therefore cannot discard the existence of variants in these genes in our LBs. Future implementations of our procedure will include a gene panel that is more specific to FA.

## 5. Conclusions

In conclusion, the deep-sequencing of saliva and plasma from FA patients can detect mutations in cancer genes that stratify FA patients into risk groups. This technology is particularly applicable to FA patients with no visible lesions. Therefore, we propose that surveillance protocols with a liquid biopsy analysis performed repeatedly, every year, should be recommended for all FA patients as the existence of mutations predictive of the future development of OPMLs and OSCCs in the absence of clinical symptoms is possible. Further similar longitudinal studies that increase the number of patients and the follow-up time will determine whether our approach allows for the earlier diagnosis of lesions or carcinomas in the oral cavity.

## Figures and Tables

**Table 1:** Fanconi anemia patient cohort characteristics.

Characteristics	Fanconi Anemia Patients (*n* = 16) *n* (%)
Age (years)	
Median	27
Range	14–46
Sex (*n*)	
Female	9 (56)
Male	7 (44)
Patients with HSCT	11 (69)
Median years from HSCT to study launch date	8.4
GVHD	4 (24)
Follow-up time (days)	
Median	719
Range	454–1118
Patients with lesions/tumors	
Oral potentially malignant lesion	6 (35)
Oral Squamous Cell Carcinoma	2 (12)

Abbreviations: HSCT: hematopoietic stem cell transplantation; GVHD: graft-versus-host-disease.

## Data Availability

The data generated during this study are available upon request to the corresponding author.

## Supplementary Materials

The following supporting information can be downloaded at: https://www.mdpi.com/article/10.3390/cancers15061871/s1, Table S1: Fanconi anemia patients’ specific characteristics, Table S2: Oncomine^TM^ Pan-Cancer Cell-Free Assay, Table S3: Sequencing performance per liquid biopsy sample in FA patients, Table S4: Non-synonymous small variants or mutations (SNVs, MNVs and INDELs) found in LBs and leukocytes, Table S5: Lesions and SCC during follow-up of FA patients.
