# Supplementary material for: Early Diagnosis of Oral Cancer and Lesions in Fanconi Anemia Patients: A Prospective and Longitudinal Study Using Saliva and Plasma
Source: Cancers (Basel). 2023 Mar 21;15(6):1871. doi: 10.3390/cancers15061871 (PMC10046988; doi:10.3390/cancers15061871)
Supplement: Supplementary file 1 [file cancers-15-01871-s001.zip › cancers-2193163-supplementary.pdf]

Table S1. Fanconi anemia patients' specific characteristics

Patient	Sex	Age[1]	HSCT[2]	Donor[3]	GVHD[4]
FA122	female	28	no	na	no
FA531	female	30	no	na	no
FA82	female	46	no	na	no
FA103	male	29	yes	mrd	yes
FA467	male	14	yes	msd	no
FA452	male	17	yes	msd	no
FA145	female	22	yes	ad	yes
FA536	female	16	yes	mud	no
FA228	male	31	yes	mud	yes
FA294	female	36	yes	msd	no
FA746	female	16	yes	msd	no
FA29	female	29	no	na	no
FA328	female	17	yes	mud	yes
FS829	male	38	no	na	no
FA374	male	17	yes	mrd	no
FA49	male	26	yes	mud	no

[1] Age at patient recruitment
[2] HSCT: hematopoietic stem cell transplantation
[3] Donor: donor type when HSCT. mrd: matched related donor; msd: matched sibling donor; mud: matched unrelated donor; ad: alternative donor; na: non applicable.
[4] GVHD: graft-versus-host-disease

Table S2. Oncomine™ Pan-Cancer Cell-Free Assay

Hotspot genes			Tumor suppressor genes	CNV genes	Gene fusions *
AKT1	*FGFR3*	*MTOR*	*APC*	*CCND1*	*ALK*
ALK	*FGFR4*	*NRAS*	*FBXW7*	*CCND2*	*BRAF*
AR	*FLT3*	*NTRK1*	*PTEN*	*CCND3*	*ERG*
ARAF	*GNA11*	*NTRK3*	*TP53*	*CDK4*	*ETV1*
BRAF	*GNAQ*	*PDGFRA*		*CDK6*	*FGFR1*
CHEK2	*GNAS*	*PIK3CA*		*EGFR*	*FGFR2*
CTNNB1	*HRAS*	*RAF1*		*ERBB2*	*FGFR3*
DDR2	*IDH1*	*RET*		*FGFR1*	*MET*
EGFR	*IDH2*	*ROS1*		*FGFR2*	*NTRK1*
ERBB2	*KIT*	*SF3B1*		*FGFR3*	*NTRK3*
ERBB3	*KRAS*	*SMAD4*		*MET*	*RET*
ESR1	*MAP2K1*	*SMO*		*MYC*	*ROS1*
FGFR1	*MAP2K2*				
FGFR2	*MET*				

• 52 genes
• Single library from DNA and RNA*
• 272 amplicons
• >900 hotspots and indels
• Extended coverage of *TP53*
• 96 fusions
• 12 CNVs
• *MET* exon 14 skipping

* RNA was not processed and gene fusions were not interrogated

Table S3. Sequencing performance per liquid biopsy sample in FA patients.

patient	collection time (per patient)	sample type	ng DNA in sample	median molecular coverage[1]	LOD (%)[2]	mapped reads[3]	on target reads (%)[3]
FA029	1	plasma	16.0	1508	0.2835	23,252,734	94.5
FA029	1	saliva	16,500	11345	0.0767	24,820,351	96.36
FA029	3	plasma	21.5	1827	0.2341	11,629,004	93.31
FA029	3	saliva	65,250	7718	0.077	17,646,973	96.44
FA049	1	plasma	18.0	970	0.4404	9,691,979	93.4
FA049	1	saliva	71,250	6143	0.0771	16,753,934	97.47
FA049	2	plasma	10.6	1274	0.3356	na	na
FA049	2	saliva	6,213	6107	0.0771	na	na
FA049	3	saliva	1,658	5863	0.0771	13,204,407	92.92
FA082	2	plasma	17.7	1806	0.2368	na	na
FA082	2	saliva	3,025	7576	0.077	na	na
FA082	4	plasma	31.8	1103	0.3876	14,061,179	91.42
FA082	4	saliva	26,500	7839	0.077	17,869,458	96.15
FA103	1	plasma	27.8	908	0.4704	10,700,829	94.19
FA103	1	saliva	35,625	8525	0.077	23,583,046	97.29
FA103	2	plasma	29.5	3915	0.1093	16,066,352	94.97
FA103	2	saliva	12,500	11106	0.0768	22,415,353	97.12
FA103	3	saliva	12,200	6576	0.0771	24,088,967	96.99
FA122	1	plasma	37.1	3787	0.1129	10,544,807	92.24
FA122	1	saliva	24,125	7298	0.077	13,981,295	95.81
FA145	1	plasma	26.5	3024	0.1414	10,997,167	93.31
FA145	1	saliva	34,750	5365	0.0797	10,815,243	97.22
FA145	2	plasma	33.6	3334	0.1283	na	na
FA145	2	saliva	11,900	5804	0.0771	23,564,390	96.84
FA145	3	saliva	6,975	5339	0.0801	11,088,276	94.8
FA145	4	saliva	23,125	4713	0.0908	10,415,095	94.13
FA145	4	leucocytes	432	3207	0.1334	12,641,714	93.77
FA228	1	plasma	19.6	940	0.4545	13,408,865	93.16
FA228	1	saliva	27,250	12623	0.0767	25,928,587	97.41
FA228	2	saliva	140,000	5836	0.0771	na	na
FA228	2	plasma	9.4	957	0.4466	na	na
FA228	3	leucocytes	246.6	4085	0.1047	18,923,515	95.63
FA228	3	plasma	10.7	1011	0.4228	na	na
FA228	3	saliva	64,370	7992	0.077	na	na
FA228	4	plasma	13.9	1622	0.2635	na	na
FA228	5	saliva	52,250	7830	0.077	19,684,271	96.84
FA294	1	plasma	10.4	633	0.6748	9,188,463	93.78
FA294	1	saliva	4,500	8605	0.077	26,520,004	97.15
FA328	1	saliva	7,417	4290	0.0997	12,232,402	97.48
FA328	1	plasma	13.5	863	0.4952	16,046,003	93.33
FA328	3	saliva	1,542	4647	0.092	18,446,600	96.01
FA374	1	saliva	20,375	6105	0.0771	26,463,807	97.7
FA374	2	saliva	6,850	13376	0.0766	na	na
FA374	3	plasma	13.6	650	0.6567	9,478,134	94.7
FA374	3	saliva	6,875	11185	0.0768	30,112,655	97.21
FA374	4	saliva	34,250	7154	0.077	19,425,740	96.77
FA452	1	saliva	4,917	4637	0.0922	20,406,290	97.72
FA452	2	saliva	12,500	3681	0.1162	17,321,375	95.86
FA452	2	plasma	11.7	548	0.7785	7,081,164	93.4
FA452	3	saliva	13,375	6097	0.0771	12,785,876	94
FA452	3	plasma	43.8	4324	0.0989	17,938,349	92.88
FA467	1	leucocytes	714	3729	0.1147	19,272,522	95.61
FA467	1	plasma	16.4	1859	0.23	na	na
FA467	1	saliva	16,375	7868	0.077	na	na
FA531	2	saliva	12,225	5072	0.0843	10,889,921	92.08
FA531	3	saliva	11,775	3053	0.1401	13,829,778	94.58
FA531	3	plasma	10.7	1311	0.3261	16,004,566	92.79
FA531	4	saliva	63,500	4103	0.1042	12,653,106	95.91
FA531	4	leucocytes	285	4520	0.0946	15,931,260	94.21
FA536	1	saliva	13,620	7096	0.0771	36,225,694	97.47
FA536	1	plasma	20.0	1079	0.396	12,515,086	92.76
FA536	2	plasma	15.5	1784	0.2396	na	na
FA536	2	saliva	4,875	10496	0.0769	na	na
FA536	3	saliva	6,325	9233	0.0769	36,319,049	96.87
FA536	4	saliva	7,925	14727	0.0765	31,401,604	97.04
FA746	1	saliva	788	2026	0.2111	13,898,152	95.23
FA746	2	saliva	3,825	6096	0.0771	30,577,592	96.99
FA746	2	plasma	285.0	3247	0.1317	23,542,906	95.81
FA829	1	saliva	55,000	10159	0.077	na	na
FA829	2	saliva	91,250	9842	0.077	29,544,169	96.8

[1] Median molecular coverage reports median number of individual interrogated DNA molecules across targets.

[2] LOD: limit of detection (median value across all targets)

[3] na: values that could not be retrieved

Table S4. Non-synonymous small variants or mutations (SNVs, MNVs and INDELs) found in LBs and leukocytes

patient	collection time (per patient)	cancer gene	AA change	MAF (%)[1]	saliva	plasma	leukocytes[2]
FA082	4	TP53	p.E180GfsTer67	0.1907		yes	
FA103	2	PDGFRA	p.A466VfsTer39	0.0955		yes	
FA122	1	TP53	p.R175H	7.2159	yes		
FA122	1	TP53	p.R175H	33.7177		yes	
FA145	1	TP53	p.V157I	0.0768	yes		
FA145	1	TP53	p.W91L	0.0777	yes		
FA145	1	TP53	p.P92A	0.0932	yes		
FA145	3	TP53	p.V157I	0.0998	yes		
FA145	2	TP53	p.V157I	0.1148		yes	
FA145	4	PDGFRA	p.A466VfsTer39	0.2536			yes
FA228	1	TP53	p.P92A	0.0837	yes		
FA228	4	TP53	p.T102P	0.2742		yes	
FA452	2	TP53	p.W146SfsTer3	0.3911	yes		
FA452	2	TP53	p.W146SfsTer3	0.8229		yes	
FA467	1	TP53	p.Q38R	0.2323		yes	
FA531	2	TP53	p.G245S	0.0726	yes		
FA531	2	IDH2	p.R140Q	0.0758	yes		
FA531	3	TP53	p.G245S	0.2911	yes		
FA531	4	IDH2	p.R140Q	0.1121			yes
FA536	4	TP53	p.P92A	0.0928	yes		
FA536	4	TP53	p.W91L	0.0986	yes		

[1] MAF: molecular allele frequency

[2] Used to discard possible mutations in saliva/plasma as origitated in white blood cells upon clonal hematopoiesis

Table S5. Lesions and SCC during follow-up of FA patients

Patient	Time order	Location	Oral Lesion	Lesion grade
FA145	1	retromolar trigone	leuko/erytroplakia	high grade displasia
	3	retromolar trigone	margin expansion from SCC	high grade displasia
FA452	1	retromolar trigone	leukoplakia	no biopsy
FA228	1	palatal mucosa	leukoplakia	low grade displasia
	1	buccal mucosa	leukoplakia	low grade displasia
	2	hard palate	leukoplakia	hyperplasia
	2	buccal mucosa	leuko/erytroplakia	non dysplasia
	3	right lip	ulcer	hyperplasia/dysplasia
	4	lip/oral mucosa	leukoplakia	low grade displasia
	5	vestibular fundus & tongue margin	leukoplakia	hyperplasia
FA103	1	tongue	leukoplakia	no biopsy
FA467	1	buccal mucosa	leukoplakia	no biopsy
FA374	1	alveolar ridge	leukoplakia	no biopsy
FA374	2	alveolar ridge & buccal mucosa	leukoplakia	no biopsy

Patient	Time order	Location	Oral Cancer	Tumor stage
FA531	1	base of tongue	SCC	pT2N0Mx p16-
FA145	2	retromolar trigone	SCC	pT1NxMx p16-
